# Protocol for the evaluation of a social franchising model to improve maternal health in Uttar Pradesh, India

**DOI:** 10.1186/s13012-015-0269-2

**Published:** 2015-05-26

**Authors:** Shreya K. Pereira, Paresh Kumar, Varun Dutt, Kaveri Haldar, Loveday Penn-Kekana, Andreia Santos, Timothy Powell-Jackson

**Affiliations:** Department of Global Health and Development, London School Hygiene and Tropical Medicine, 15-17 Tavistock Place, London, WC1H 9SH UK; Sambodhi Research and Communications Limited, C-126, Sector-2, Noida, Uttar Pradesh India; Department of Infectious Disease Epidemiology, London School Hygiene and Tropical Medicine, Keppel Street, London, WC1E 7HT UK

**Keywords:** Social franchising, Impact evaluation, India, Study protocol

## Abstract

**Background:**

Social franchising is the fastest growing market-based approach to organising and improving the quality of care in the private sector of low- and middle-income countries, but there is limited evidence on its impact and cost-effectiveness. The “Sky” social franchise model was introduced in the Indian state of Uttar Pradesh in late 2013.

**Methods/design:**

Difference-in-difference methods will be used to estimate the impact of the social franchise programme on the quality and coverage of health services along the continuum of care for reproductive, maternal and newborn health. Comparison clusters will be selected to be as similar as possible to intervention clusters using nearest neighbour matching methods. Two rounds of data will be collected from a household survey of 3600 women with a birth in the last 2 years and a survey of 450 health providers in the same localities. To capture the full range of effects, 59 study outcomes have been specified and then grouped into conceptually similar domains. Methods to account for multiple inferences will be used based on the pre-specified grouping of outcomes. A process evaluation will seek to understand the scale of the social franchise network, the extent to which various components of the programme are implemented and how impacts are achieved. An economic evaluation will measure the costs of setting up, maintaining and running the social franchise as well as the cost-effectiveness and financial sustainability of the programme.

**Discussion:**

There is a dearth of evidence demonstrating whether market-based approaches such as social franchising can improve care in the private sector. This evaluation will provide rigorous evidence on whether an innovative model of social franchising can contribute to better population health in a low-income setting.

**Electronic supplementary material:**

The online version of this article (doi:10.1186/s13012-015-0269-2) contains supplementary material, which is available to authorized users.

## Background

Over the past few decades, India’s maternal mortality ratio has declined substantially from 437 deaths per 100,000 live births in 1992–1993 to 178 deaths per 100,000 live births in 2010–2012 [1, 2]. Despite these improvements, the current state of maternal and child health in India requires urgent attention. India remains the largest contributor to the global burden of maternal deaths, accounting for nearly a quarter of all maternal deaths worldwide [3]. One of the most high profile responses of the Government of India has been to encourage facility births by providing cash incentives to women through the Janani Suraksha Yojana (JSY) scheme. Studies show that the programme has been effective in increasing utilisation of government maternal health services even if the evidence on mortality is contested [4, 5]. However, there are concerns about the public sector and its capacity to meet the increased demand for institutional deliveries. Whether the resources of the private sector should be harnessed to improve maternal health, and other aspects of health, is at the forefront of ongoing debates [6].

India’s private health sector is extensive and incredibly diverse. It ranges from sophisticated tertiary hospitals providing medical care of an international standard to unqualified rural health providers and alternative systems of medicine. The majority of registered doctors work in the private sector, and it is often the first point of contact for a substantial proportion of the population [7–9]. In Uttar Pradesh, the setting of this study, 31 % of all facility births are in the private sector [10]. Although no worse than the public sector, studies of the private sector in India document poor quality of primary health care and potentially harmful practices [11].

Despite widespread consensus on the growing presence and role of the private sector in low- and middle-income countries, there is limited evidence on the most effective strategies to improve the quality of services [12, 13]. Regulating the quality of the private sector given its size and diversity has proved enormously challenging for the government, and alternative strategies to raise standards must be sought. Innovative approaches currently being used to tackle and institutionalise quality improvement include accreditation [14], contracting out clinical services [15], vouchers [16], and social franchising [17].

Social franchising is the fastest growing market-based approach to organising and improving the quality of care in the private sector in low- and middle-income countries [18]. In 2013, 83 franchises were largely operating in Sub-Saharan Africa and Asia reaching nearly 20 million patients [19]. Social franchises are networks of private providers, operating under contracts with a common agency and providing standardised products and services under a single brand. Social franchises typically have five programmatic goals: quality, health impact, equity, cost-effectiveness and market expansion. Franchise models that link with and encourage referrals between the public and private sector may help reduce health market fragmentation and improve quality of care. Common franchise elements include demand- and supply-side components relating to contract design, training, supervision, branding and advertising.

This study protocol describes the methods to be used in an evaluation of the Sky social franchising model in Uttar Pradesh. The aim of the social franchise model is to increase access to and use of basic obstetric care, emergency obstetric care and family planning services. The evaluation will draw on quantitative and qualitative methods to address three study objectives: (1) to estimate the impact of the social franchising model on the quality and coverage of health services along the continuum of care for reproductive, maternal and newborn health; (2) to understand the scale of the social franchise network, the extent to which various components of the programme are implemented and how impacts are achieved; and (3) to establish the cost-effectiveness and financial sustainability of the programme.

### Evidence on social franchising

Our review of the evidence draws on three recent systematic reviews of social franchising in health [17, 18, 20]. The majority of the social franchise programmes focus on reproductive services and family planning products, which together account for a large proportion of the literature on the topic [18, 20]. Before examining the empirical evidence, it is important to note that the methodological rigour of studies on social franchising in low- and middle-income countries is poor. This is demonstrated by the fact that the most thorough review of social franchising, published in the Cochrane library, found no studies eligible for inclusion despite the fact that inclusion criteria were broad enough to permit a range of quasi-experimental methods [17]. None of the reviews uncovered any evidence on the health impact or cost-effectiveness of social franchising.

In a second review, studies of clinical social franchise programmes were included if they provided data on at least one outcome related to quality, health impact, equity, cost-effectiveness and market expansion [18]. Quasi-experimental and qualitative studies were not excluded. The authors included 23 studies whose overall quality was regarded as low. The review found limited and mixed evidence on impact. Social franchising was found to increase client volume and service utilisation, but there was no evidence on the ability of social franchising to expand the availability of health services in currently underserved areas. Over half of the studies measured some aspect of quality but always in relation to family planning services and rarely in a comprehensive manner. A study in Pakistan and Ethiopia found that franchises were of equivalent or lower quality than public clinics but higher quality than non-franchised private providers. In Nepal, both franchised and non-franchised clinics showed similarly poor facility quality.

A third review of social franchising included 15 studies that examined the relationship between franchising and outcomes [20]. Around half focused on quality and utilisation, and a few considered results for providers, client loyalty, client volumes and efficiency. Reproductive health/family planning services research were well represented; other sectors investigated were pharmacy and tuberculosis care. The authors found that franchising is predominantly positively associated with client volumes, physical accessibility and some types of quality, but findings regarding utilisation, customer loyalty and efficiency were mixed. The methodological quality of studies was found to be poor.

In summary, the current scientific evidence and body of knowledge on the impact of social franchising, or on the sustainability of social franchising as a long-term alternative to the public sector, suggest that generalisations about the value of franchising are difficult to make. There is some evidence on the ability of clinical social franchising to increase patient volume and some aspects of quality of care. However, in general, the quality of evidence is sufficiently poor and variation in the types of social franchising models tested so wide that no firm conclusions can be drawn about whether and how social franchises affect service delivery.

### Social franchise model

The Sky franchise network includes providers at various levels. SkyCare is the lowest level of the network and consists of informal rural health providers who are typically medically unqualified. SkyCare providers pay a franchise joining fee to the franchisor and receive signage, posters, training manuals and the ability to phone into a central medical facility. The franchisor maintains the central medical facility by employing qualified doctors to conduct remote medical consultations. SkyCare franchisees are given financial incentives by the network to make antenatal care referrals to SkyHealth franchisees. The second level of the network is SkyHealth. These providers are typically qualified traditional medical practitioners trained to provide Ayurvedic, Yoga & Naturopathy, Unani, Siddha and Homoeopathic care (AYUSH). SkyHealth offers telemedicine services and receive financial incentives for completing three antenatal care consultations with a client. At the highest level, nine franchised clinics and three franchisor-owned clinics are staffed by physicians to provide safe delivery and emergency obstetric care.

The programme provides clinical training to private and public sector health providers. The franchisor trains SkyCare in how to conduct mobile phone consultations. SkyHealth is trained to provide antenatal care, recognise and stabilise pregnancy complications, facilitate timely referrals and provide family planning methods and postpartum contraception counselling. Providers from franchised clinics receive training on national and international guidelines to provide emergency obstetric care, general family planning and postpartum intrauterine devices. Public sector providers with the remit of dealing with emergency obstetric cases also receive clinical training in order to manage linkages and referrals from the private sector. Training in the public sector extends to Accredited Social Health Activist (ASHA) working at the community level. An additional file details the training programme by type of provider [see Additional file 1].

The social franchise model takes a total market approach in the sense that it seeks to develop closer links to, and strengthening of, the public sector. The major components of the programme are summarised in Table 1, using an adaptation of the Centre for Health Market Innovations framework for characterising health programmes [21]. Components fall into five major approaches: organising delivery, regulating performance, financing care, changing behaviours and enhancing processes. These approaches include both demand- and supply-side activities to influence health care-seeking behaviours and the quality of healthcare provision. Demand-side activities include incentivising rural health providers for referrals, brand creation, price subsidies for clients below the poverty line and advertising of franchise services. A major demand-side activity of the programme is social marketing, in which the franchisor distributes their own branded medicines—SkyMeds—via a network of shops, pharmacies and franchisees. SkyCare and SkyHealth providers are encouraged to sell SkyMeds, though it is optional. Supply-side activities include clinical training for providers, telemedicine and mobile technologies and the introduction of innovations such as the non-pneumatic anti-shock garment to stabilise women with heavy bleeding in both private and government facilities.

**Table 1:** Components of the social franchising programme

Organising delivery	Programmes that reduce fragmentation and informality of health care delivery and that may enable financing, regulation, training and new business models	
Franchise	A group of providers that operates under the same brand but where outlets are operator-owned and services are standardised by a central franchisor	SkyCare/SkyHealth (stand-alone franchises); franchise clinic/franchise diagnostic (fractional franchises)
Chain	A group of providers that operates under the same brand but where operators are paid employees of a sponsoring organisation	Three franchisor-owned clinics (also called mini-clinics)
Network	A group of providers that are loosely joined to deliver services to specific population groups. Each provider is a separate entity and retains its own branding. Membership in the network may entitle the provider to payments, patient volume, central services or training	Franchisees are linked to a network of shops selling drugs which receive socially marketed products
Regulating performance	Programmes that set standards and enforce or incentivize higher quality care or increased access for target populations	
Quality enforcement/monitoring	Programmes that mandate specific clinical practice guidelines, and/or monitor providers over time to ensure quality	Monitoring and supervision of quality standards in franchisees, exit surveys and encourage feedback from competitors
Price regulation	Programmes or regulations that specify prices that must be charged to users for services	Fixed prices for below the poverty line clients at Sky Centres; fixed prices for franchised services at franchised clinics
Financing care	Programmes that mobilise funds for health care and align provider incentives to increase access for targeted groups of patients or to support select health interventions	
Links to government health financing mechanisms	Initiatives that link private providers to existing government health financing mechanisms that can contract and reimburse private providers for care provided to specified patient groups	Plan to facilitate linking franchisees and beneficiaries to government cash incentive and insurance schemes. Training of community health workers to link with government schemes
Cross subsidisation	Programmes that charge full-fees for services to patients that are able to afford them and use the profits to subsidise services for the poor	Subsidies for telemedicine for clients below the poverty line off-set to some degree by franchise fee paid per client above the poverty line
Changing behaviours	Programmes designed to change the behaviour of individuals involved in health care transactions	
Social marketing	Programmes that aim to change consumer care-seeking behaviours through marketing/advertisement techniques, with or without a branded and/or subsidised product	Branding, advertising, SMS messages, provision of SkyMeds
Community health workers	Programmes that use community health workers to generate demand for products or services	Government community health workers refer women to public and franchised facilities
Provider training	Programmes that seek to improve the quality and/or efficiency of services by training health care workers and/or building the internal capacity of organisations	Training of SkyCentre staff, franchise clinic staff, community health workers and public sector staff. Sky centre staff also trained on telemedicine technology
Other health awareness/education	Programmes that create social awareness and educate the public about specific health topics such as disease prevention and treatment, healthy behaviours, correct use of pharmaceuticals, etc.	Community system to give health messages
Organising delivery	Programmes that reduce fragmentation and informality of health care delivery and that may enable financing, regulation, training and new business models	
Enhancing processes	Processes, technologies, or products that facilitate increased efficiency, lower costs, higher quality, and/or improved access	
Information and communications technology	Programmes that utilise technology to enable remotely delivered care, communication and exchange of medical information (e.g. telemedicine, call centre, cell phone technology, biometric system, etc.).	Cell phone/smartphone/tablet/telemedicine services through franchisees, including remote diagnostics
Innovative operational processes	Programmes that improve quality, reduce costs or enhance efficiency of services through new business or care processes (e.g. high-volume/low-cost operational models, process standardization).	Telemedicine; getting auxiliary nurse midwives to insert intrauterine devices in rural areas
Mobile health	Programmes that utilise various models of transportation to deliver services to rural and remote populations. (e.g. ambulance services, health worker transport, travelling clinics/products, etc.)	May have Sky ambulance and link to “108” ambulance
Supply chain enhancements	Programmes that reduce costs and improve efficiency of supply chains that move medical products from manufacturer to retailer	Last mile outriders (SkyMeds and diagnostics)
Innovative medical products and equipment	Programmes that design, manufacture and sell new products such as rapid testing kits, nutritional supplements or other medical supplies that reduce costs, improve quality or enable remote care	Non-pneumatic anti-shock garment; stabilisation procedures at lower levels; remote diagnostics; safe delivery kits

### Theory of change

A theory of change for the social franchising programme was developed in collaboration with the implementing franchisor in December 2013. The results chain of the Sky social franchise model was used for this purpose and is detailed in an additional file [see Additional file 2]. It shows the sequence of inputs, activities and outputs that are expected to improve outcomes. The results chain is clearly a naïve simplification of reality, but it nevertheless provides a useful framework for understanding how the programme is intended to work as originally designed.

We highlight here the most important pathways that are critical to generating the intended impacts. First, health providers are willing to join the network and expand the range of services they offer to include reproductive and maternal health services. Second, the branding of social franchisees provides a signal of quality that increases patient demand for their services. Third, the monitoring of standards and the prospect of better business performance encourage health providers to improve their quality of care. Fourth, training and the provision of IT such as telemedicine increase skills and knowledge, ultimately leading to better quality of care and appropriate referrals.

Several key assumptions underpin the programme’s success. All else equal, basic economic theory suggests that increasing the quality of a service will raise consumer demand. In healthcare, however, information problems are pervasive and patients may not in fact be able to evaluate the quality of care they received, at least in terms of aspects of care that matter for health [22–24]. The extrinsic incentives to improve quality may therefore not be strong. Another important assumption is that lack of provider knowledge and skills are binding constraints to delivering quality healthcare. The evidence here is somewhat mixed. A meta-analysis of studies in low- and middle-income countries shows that training has a modest effect on provider practice [25]. In India, the qualifications of the provider matter for quality but not as much as expected [26].

## Methods

### Study setting

Uttar Pradesh is India’s fourth largest and most populous state with approximately 199.8 million people living in 18 divisions and 75 districts. If Uttar Pradesh were a country, it would be the fifth largest in the world in terms of population. The three districts in which the social franchising network is located have a population of 8.1 million and vary considerably in terms of demographic and health indicators (see Additional file 3). Kanpur Nagar is predominant urban, with higher literacy and lower mortality than the state average. By contrast, Kannauj and Kanpur Dehat are more typical of the state as a whole. Largely rural, they have poor literacy and high rates of maternal and child mortality that are comparable with the less developed countries in the world.

Across the continuum of care, large discrepancies in maternal and child health indicators are observed between the three districts [see Additional file 3]. For example, coverage of at least three visits of antenatal care in Kanpur Nagar is the highest at 51 % compared to 15 and 32 % in Kannauj and Kanpur Dehat, respectively. Despite government schemes to improve rates of institutional births, 54 % of deliveries occur at home in Uttar Pradesh (57 % in Kannauj, 40 % in Kanpur Nagar and 52 % in Kanpur Dehat). Of the home deliveries, 11 %, 53 % and 29 % were conducted by skilled health personnel in Kannauj, Kanpur Nagar and Kanpur Dehat, respectively.

### Study design

**Fig. 1:**
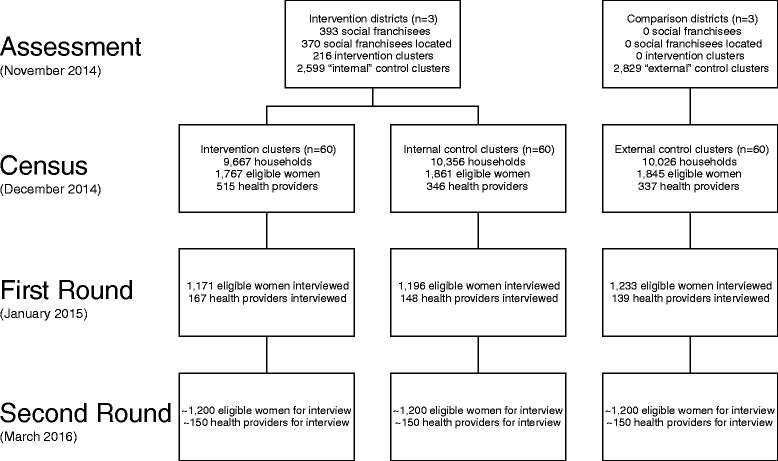
Study design and data collection

The impact study is designed as a prospective controlled before and after study in which the comparison group comprises matched areas both within the intervention districts and in neighbouring districts where social franchising is not introduced. The overall design is shown in Fig. 1. The primary sampling unit for much of the data collection is a cluster, defined as a ward (urban) or a village (rural) according to the most recent census. The impact evaluation involves the selection of study clusters to form three arms. Group A contains clusters with a social franchisee in the three intervention districts. Group B comprises clusters with no social franchisee in the same three districts. Group C is taken from neighbouring districts that do not have any social franchise network operating within them.

**Fig. 2:**
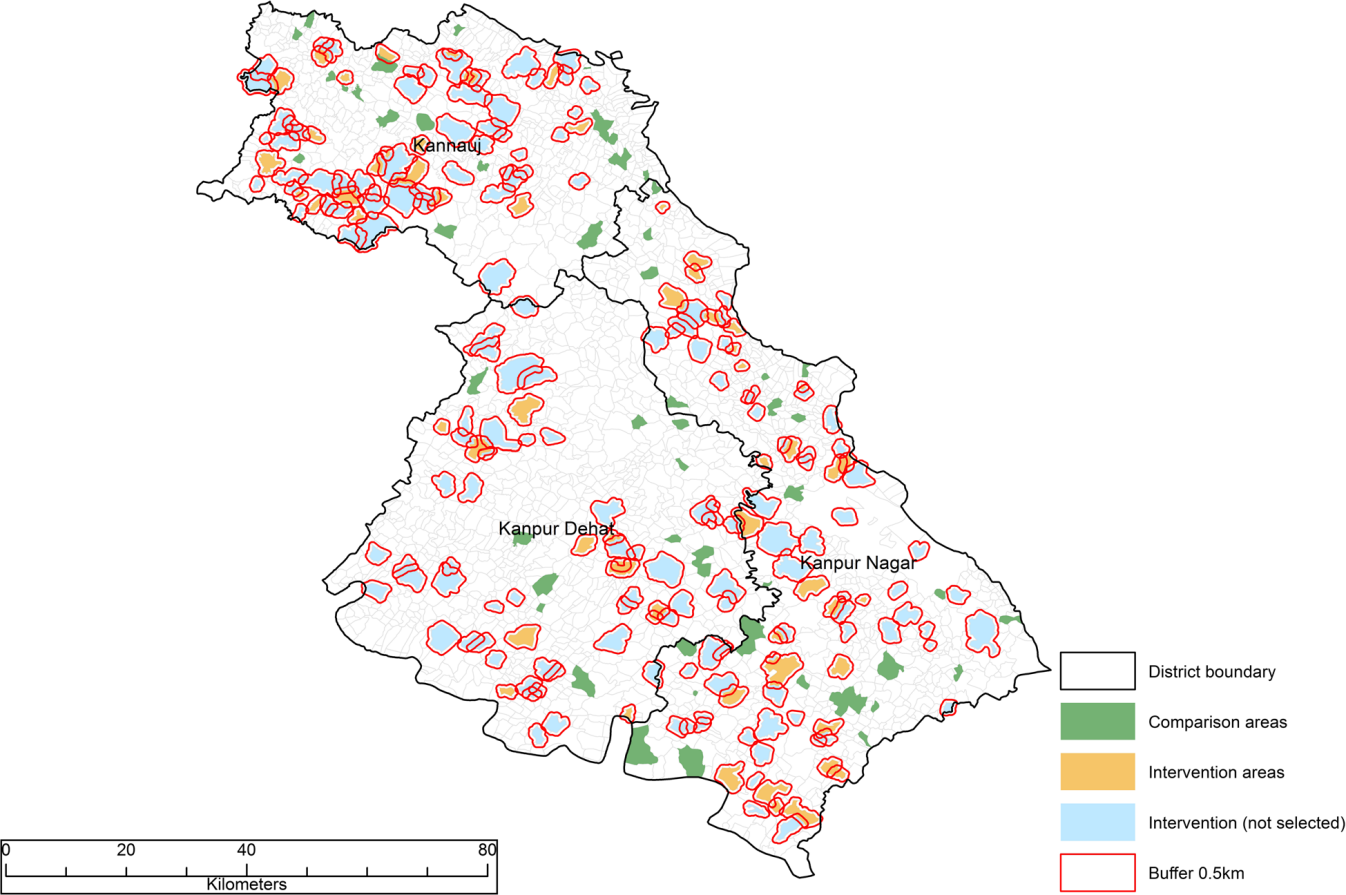
Map of study clusters in the three intervention districts

The selection of study areas was done 14 months after the first health providers were contracted at which time there were 50 SkyHealth and 343 SkyCare providers in the social franchise network. The selection of study clusters proceeds according to the following steps. First, we link every social franchisee to the census area in which it is located and select, at random, 60 intervention clusters (Group A). Second, we select similar controls by matching without replacement the intervention clusters to 60 comparison areas within the same three districts (Group B) [27]. As can be seen in Fig. 2, we impose a buffer zone of 0.5 km around intervention clusters to limit problems of contamination. We perform exact matching on district and urban residences and then within each strata, select pairs of clusters (nearest neighbour) with the smallest distance based on a Mahalanobis metric that is computed using census data on total population, % under 6 years, % females under 6 years, % literate females, % scheduled tribe, % scheduled caste, % cultivator and % “other” workers. Finally, we perform the same matching procedure to select 60 comparison areas in neighbouring districts (Group C).

The selection of study clusters provides variation in the social franchise that facilitates identification of its impact. Variation over time is generated in two ways. The 2-year recall period of the household survey means that we have almost 12 months of baseline data even in areas where social franchising is introduced. Moreover, we anticipate that over time, some of the Group B study clusters will become intervention areas as the social franchise network expands generating further variation over time. Geographical variation in the placement of the social franchise is generated by our selection of comparison areas.

### Data collection

The evaluation relies primarily on several tools that are administered over two rounds of data collection: a household survey of women who recently gave birth and a health provider survey. The first round of data collection was in January 2015, and the second round is planned for March 2016. This means the impact of the social franchise intervention is assessed approximately 2 years after its start. Information on the precise timing of the introduction of social franchising in each cluster is based on administrative data provided by the franchisor triangulated with responses to the health provider survey. It is worth noting that the research outlined in this protocol is closely coordinated with several other data collection activities—direct observations of births and a case study of three social franchise models that will provide additional insights.

The household survey is administered to women as a cross-section at two points in time and serves as the main source of data on our study outcomes. Eligible respondents include all women who gave birth in the previous 24 months (first round) or 18 months (second round), including those who had a stillbirth or whose child died since birth. Eligible women are identified through a census of households, conducted 1 month before the household survey. Every member of the household is listed and then, for women aged 15 to 49 years, a series of questions probe whether she gave birth to a baby that was born alive, born dead or lost before birth. Using this sampling frame, a maximum of 23 eligible women in each cluster are randomly selected for interview. The household survey tool includes the following modules: (1) household listing, (2) general healthcare interactions, (3) household characteristics, (4) wellbeing of husband, (5) pregnancy history, (6) family planning and antenatal care, (7) delivery and postnatal care, (8) child health, (9) interactions with community health workers, (10) information and perceptions of healthcare, and (11) wellbeing, mental health and physical health.

The health provider survey is administered at the same time as the household survey within the same communities. The sampling frame is generated from a census of all health providers within the study clusters conducted 1 month prior to the health provider survey. The census records the type of health provider and its geographic coordinates. For the purposes of the census, we define a health provider as any institution or individual whose primary purpose is to provide healthcare. We exclude drug sellers. Using this list, we randomly select for interview one private health provider (social franchisee in intervention clusters), one government health provider and one accredited social health activist in each cluster. The health provider survey tool includes the following modules: (1) health facility characteristics, (2) key health worker characteristics, (3) reproductive, maternal and newborn services, (4) maternal health knowledge, (5) maternal health practice, (6) motivation, (7) social franchise and business practices, and (8) experience of social franchise network.

### Outcomes

The impact of the social franchise model is assessed using a comprehensive set of pre-specified outcomes that are measured using the household data. An extensive list of outcomes, 59 indicators in total, is shown in Tables 2, 3 and 4. They cover the continuum of care from antenatal care through postnatal family planning and include various types of indicators including healthcare utilisation, process of care, healthy behaviour, patient experience, patient information and financial strain. The study outcomes are organised according to conceptually similar groups that are required when implementing methods to deal with many outcomes.

**Table 2:** Antenatal outcomes of the impact evaluation by domain

Indicator	Type of indicator
1. ANC utilisation	
Received at least three ANC visits (%)	Use of healthcare
Received ANC visit in first trimester (%)	Use of healthcare
Number of ANC consultations (visits)	Use of healthcare
Received visit from ASHA during pregnancy (%)	Use of healthcare
2. ANC content of care	
Fully immunised with tetanus toxoid (%)	Process of care
Received iron supplementation during pregnancy (%)	Process of care
Took iron supplementation during pregnancy for at least 100 days (%)	Process of care
Received test results for syphilis received (%)	Process of care
Abdominal examination during ANC (%)	Process of care
Received a drug for intestinal worms during pregnancy (%)	Process of care
Received a drug to prevent malaria (%)	Process of care
Multiple birth pregnancy detected during ANC (%)	Process of care
ANC content of care score of six items (index 0 to 1)	Process of care
3. ANC knowledge and preparedness	
Mother knowledge of pregnancy complications (index 0 to 1)	Patient knowledge
Mother knowledge of signs of delivery complications (index 0 to 1)	Patient knowledge
Birth preparedness (financial, transport, blood donor, attendant, safe delivery kit) (index 0 to 1)	Healthy behaviour

**Table 3:** Intrapartum care outcomes of the impact evaluation by domain

Indicator	Type of indicator
1. Delivery care utilisation	
Gave birth in a health facility (%)	Use of healthcare
Gave birth with a doctor, nurse or midwife (%)	Use of healthcare
Had a caesarean section (%)	Use of healthcare
2. Recommended delivery care practices	
Delivery attendant used gloves (%)	Process of care
Delivery attendant washed hands with soap (%)	Process of care
Woman had her BP measured (%)	Process of care
Mobility during labour (%)	Process of care
Oral fluids during labour (%)	Process of care
Heart rate of baby monitored with intermittent or continuous auscultation (%)	Process of care
Use of anti-shock garment (%)	Process of care
3. Harmful or ineffective delivery care practices	
Shaving pubic hair (%)	Process of care
Enema given (%)	Process of care
Lithotomy position during labour (%)	Process of care
Intravenous fluids during labour (%)	Process of care
4. Delivery care practices frequently over used	
Urinary catheter (%)	Process of care
Pain control by epidural analgesia (%)	Process of care
Oxytocin augmentation (%)	Process of care
Episiotomy (%)	Process of care
5. Disrespect and abuse	
Support during labour (%)	Patient experience
Medical procedure performed without consent (%)	Patient experience
Shouted, scolded or humiliated by health worker (%)	Patient experience
Slapped, pinched or hit by health worker (%)	Patient experience
Gave birth with privacy (%)	Patient experience
Refused care for inability to pay (%)	Patient experience
Kept in facility for inability to pay (%)	Patient experience
Felt disrespected or abused during facility stay (%)	Patient experience
6. Economic consequences	
Out-of-pocket spending on delivery care (NRS)	Financial strain
Borrowed money to pay for delivery care (%)	Financial strain
Household in debt to pay for delivery care (%)	Financial strain
Received JSY cash incentive (%)	Financial strain

**Table 4:** Postpartum and newborn outcomes of the impact evaluation by domain

Indicator	Type of indicator
1. Postpartum care	
Received postpartum care within 48 h of birth (%)	Use of healthcare
Newborn received postnatal care within 48 h of birth (%)	Use of healthcare
2. Newborn content of care	
Clean cord care (clean instrument to cut and tie the cord, and nothing put on cord) (%)	Process of care
Thermal care (immediate drying, wrapping, skin to skin and delayed bathing) (%)	Process of care
Baby weighed at birth (%)	Process of care
Baby registered and received certificate (%)	Process of care
3. Neonatal health	
Neonatal mortality (per 1000 live births)	Health outcome
One-day mortality (per 1000 live births)	Health outcome
Birth weight (kg)	Health outcome
4. Breastfeeding	
Immediate breastfeeding within 1 h of birth (%)	Healthy behaviour
Colostrum given to baby (%)	Healthy behaviour
Exclusive breastfeeding for 3 days (%)	Healthy behaviour
5. Family planning	
Modern contraceptive use at 3 months postpartum (%)	Use of healthcare

In contrast to standard surveys on maternal and child health in India [28, 29], we seek to measure a range of intrapartum care practices that may be affected by the social franchise, given its focus on standards. We get at the issue of quality of care by collecting information on recommended delivery care practices, harmful or ineffective practices, frequently over-used practices and disrespect and abuse indicators [30–33]. The study will also collect data on a large range of characteristics of the mother and her household. These data are used to control for potential confounding and increase efficiency of our estimates. They are also important in examining the equity impact of the project—to see which socioeconomic segments of the population benefits most from the social franchise.

### Sample size calculations

Household sample size calculations are based on an endline comparison of two groups (intervention versus control), using the proportion of women giving birth in a health facility as the primary outcome. On the basis of an observed institutional delivery rate of 50 % at baseline [10] and an assumed coefficient of variation of 0.2, a sample size of 60 intervention and 60 control clusters with a total of 20 women in each cluster are estimated to provide 80 % power to detect an 8 percentage point increase in the rate of institutional deliveries in the intervention group compared with the control at 5 % level of significance. Assuming a coefficient of variation of 0.1 reduces the detectable difference to 6 percentage points. It is anticipated that the study will have power to detect smaller differences once the analysis controls for covariates and utilises data from two survey rounds.

### Empirical analysis

The impact evaluation relies primarily on the household data. We use a difference-in-difference strategy to estimate impacts [34]. This involves a comparison of changes in the outcomes over time between the intervention and the comparison groups. The analysis exploits the longitudinal nature of the data generated by the recall period used in the two rounds of the household survey and information on the precise timing of the introduction of social franchising in each study area. Specifically, using individual level data, we regress each outcome on a dummy variable indicating whether social franchising has been introduced in the area at the time of birth, area fixed effects and quarter year fixed effects. Unadjusted estimates are reported as well as those that adjust for household characteristics. Controls for household characteristics include below the poverty line status, urban residence, religion, ethnicity, maternal education, parity, multiple birth and the recall period. We cluster the standard errors at the area level.

We test whether the social franchising model has an effect in two ways: the first analysis compares intervention areas with the two sets of comparison areas pooled together, and the second analysis compares the intervention areas with the comparison areas in adjoining districts without social franchising. The latter may arguably be less prone to selection bias since comparison areas in neighbouring districts are beyond the geographical reach of the project and may offer a more credible counterfactual. If the social franchising model is found to have an effect on any of the main outcomes, we conduct subgroup analyses with respect to below the poverty line status, maternal education and caste. Finally, to assess the so-called parallel trends assumption that underpins the difference-in-difference approach, we exploit the recall period in the household data to verify that trends in each of the outcomes are similar between the three study arms before the introduction of the social franchising model. Evidence of diverging pre-trends would be a cause for concern. Baseline outcomes and characteristics of women are also summarised for each study arm with continuous variables presented as mean (standard deviation) and categorical variables by frequencies (percentage). An additional file describes the empirical strategy of the impact evaluation in further detail [see Additional file 4].

The presence of multiple outcomes leads to the risk of arbitrarily selecting statistically significant outcomes where high values of test statistics arise by chance. Testing each hypothesis one at a time with a fixed significance increases the probability of a type-I error exponentially as the number of outcomes tested grows. We deal with multiple outcomes using several procedures that are implemented for conceptually similar groups of outcomes listed in Tables 2, 3 and 4 [35, 36]. First, we present standardised treatment effects by creating an index for multiple outcomes within each domain and testing for an effect on the index. Implicitly, this weighs each outcome the same within a domain. Second, we present family-wise *p* values adjusted to account for the multiple outcomes within a domain using the free step-down resampling method of Westfall and Young [37].

### Process evaluation

The process evaluation is intended to complement the impact evaluation. Indeed, it will run in parallel and draw on some of the same data sources. We will develop and critically assess a logic model of the project, mapping the pathways and intended effects of each component. We will next describe how the social franchise model evolves and the extent to which various components of the project are implemented on the ground. The process evaluation will then examine the factors that influence private providers’ decision to join the social franchise network. Finally, it will seek to understand how, if at all, the project influenced household decisions about healthcare and the behaviour of health providers.

Quantitative process measures will be collected to understand the extent of implementation, fidelity and scale (Table 5). These will focus on a number of different dimensions of implementation that map closely onto the various components within the project: uptake of social franchising; training of the health providers; information, branding and advertising; interactions with health workers in the social franchise network; and monitoring and feedback on quality and standards.

**Table 5:** Process measures

Indicator	Survey tool
1. Uptake of social franchising	
Proportion of private health providers who join network (%)	Health provider census
Proportion of providers who left network in past year (%)	Health provider survey
Proportion of providers purchasing and selling SkyMeds (%)	Health provider survey
2. Training	
Proportion of social franchisees that have received clinical training (%)	Health provider survey
Proportion of social franchisees that have received training in use of technology (%)	Health provider survey
3. Information and marketing	
Proportion of women who have ever heard of Sky social franchise network (%)	Household survey
Proportion of social franchisees that have been branded (%)	Health provider survey
4. Contacts with health workers	
Proportion of women who had any contact with ASHAs during pregnancy (%)	Household survey
Proportion of individuals who have used telemedicine in past 6 months (%)	Household survey
5. Monitoring and feedback	
Proportion of social franchisees that have received supervision visits past 6 months (%)	Health provider survey
Proportion of social franchisees that have received feedback on quality past 6 months (%)	Health provider survey

Qualitative data collection will focus on understanding the process of implementation and how the social franchise model leads to impact. A 6-month period of intensive data collection using ethnographic methods will provide insight into the impact of the social franchise model on the dynamics of health care provision and health seeking behaviour at the village level and provide an important understanding of the context in which the intervention operates [38, 39]. The ethnographic work will seek to understand the intervention as implemented on the ground, the factors influencing providers’ decisions to join the franchise, the influence of the intervention on provider behaviour and stakeholder perceptions of the various providers and components involved in the Sky social franchise. As the ethnographic research process progresses, new hypotheses and questions will develop as new insights occur with increasing familiarity with the context [40]. Participant observation will be carried out by experienced anthropologists who speak the local language. Researchers will keep detailed field notes of informal observations and everyday conversations. If an informant provides more detailed information or partakes in a long discussion, the field worker will ask to digitally record the interview. Field notes and audio recordings of discussions will be transcribed in the original language and then translated.

The sample of villages selected will be a convenience sample informed by the results of the first round of quantitative data collection and drawn from the 60 clusters with a social franchise provider. The ethnographic research will take place in three broad locations, covering the catchment area of SkyHealth providers and their nearby SkyCare providers. Field notes will be double coded by the ethnographers. Preliminary findings and reflections from the ethnographic research will be fed back to the research participants through community meetings in each of the localities as a form of validation.

The second level of qualitative process evaluation will take place through repeat in-depth interviews. A document review and in-depth interviews with franchisor staff will be used to understand the process of implementation as well as the development of the project. Two rounds of interviews will take place with 10 members of staff at various levels in the organisations. In-depth interviews with senior staff will include topics and questions that facilitate understanding of the decision-making behind the intervention and the key factors that shaped its design. Interviews with field staff will include questions about the experience of implementation, the process of engaging with providers and adaptations to the project over time. A context record, which documents information that may impact the implementation, the mechanisms of change and the outcomes under measurement, will be developed. This exercise will be undertaken every 6 months, using short interviews to gather information about any developments, events, setbacks and news that may have impacted implementation of the project.

### Economic evaluation

Micro-costing methods are used to estimate the financial and economic costs of setting up, maintaining and running the social franchise. Micro-costing methods using a bottom-up approach that record resource utilisation at the individual service level are employed to assess the cost of services [41, 42]. Three levels of costs are assessed: (i) costs incurred by the franchisor to plan, initiate and run the social franchise; (ii) costs of activities supporting the social franchise; and (iii) costs to the franchisees of participating in the network and providing the franchise services they offer. Data are obtained through administrative records, interviews with the franchisor, interviews with franchisees and informal observations. Costs are then classified according to: (i) start-up, defined as the initial costs related to the set-up of the social franchise; (ii) capital, defined as the costs of inputs that last for more than 1 year, to be annualised using standard methods [43]; and (iii) recurrent, defined as the costs of inputs that are incurred on a regular basis.

Effectiveness data is drawn from the impact evaluation. Since the study is not powered to measure the impact on mortality, it is necessary to model from the multiple study outcomes to the final health outcomes of deaths and disability adjusted life years (DALY) averted. This is based on a decision-tree model to be developed in light of a review of existing modelling tools such as the Lives Saved Tool (LiST) and Impact 2 [44, 45]. Cost-effectiveness ratios are presented as the cost per death averted and cost per DALY, comparing the situation with and without the social franchising programme. A number of commonly used thresholds are used for assessing whether the results can be considered “cost-effective” [46, 47]. A probabilistic sensitivity analysis using Monte Carlo simulation is conducted to test the effect of uncertainties across model parameters [48].

### Research ethics and data management

The evaluation study has been approved by the Public Healthcare Society (PHS) Ethics Review Board in India and the London School of Hygiene & Tropical Medicine in the UK. The study design has also received government clearance from the National Health Mission in the State of Uttar Pradesh.

Informed consent is obtained before administering all surveys. Information sheets are read and given to respondents and written or verbal consent is sought prior to interview. The research activities are unlikely to cause any harm since they involve no invasive procedures or examinations. In this respect, it is important to note that the research team are external; they have no responsibility for the implementation of the project or for the services delivered by health providers in the network. The research activities involving data collection through the household survey and health provider survey are not anticipated to cause any harm. There will be no direct benefit to the study participants. The main cost will be the time given by the interviewees. Some of the interviews with households will involve women whose baby may have recently died. The field workers will be trained to deal with such cases sensitively. The household survey includes a mental health screening questionnaire, known as the K10. In some instances, women with severe depression or mental health disorders may be identified through the use of this tool. In such circumstances, researchers are trained to facilitate referral of the individual to an appropriate source of care that is closest to where the woman lives.

Data from the household and health provider surveys are collected through computer-assisted personal interviews. To the extent possible, privacy is maintained during interviews with participants. The study makes every effort to minimise the risk of breaches of confidentiality, particularly in relation to data management and the linking of datasets. The research intends to link data from different sources at the cluster level. This can only be done by the principal investigators at the time of analysis. The quantitative data are to be made publicly available at the end of the project through an established data repository. The data will not contain any global positioning system (GPS) information, identifiers or names that would allow identification of an individual or cluster. In-depth interviews will be conducted in private to maintain confidentiality. Qualitative data are to be securely kept. Audio files are downloaded onto a password protected computer. Data recorded on paper and audio files will be destroyed after the data are analysed and results are reported. In the reporting of the qualitative data, quotations are anonymised such that it is not possible to identify the individual.

## Discussion

The social franchising model in Uttar Pradesh seeks to increase access to and use of basic obstetric care, emergency obstetric care and family planning services. The approach is novel in its focus on maternal health services, its effort to engage with low level and, in some cases, informal healthcare providers and its use of technology such as telemedicine. Whether such an approach to social franchising represents the way forward to improving utilisation and quality of maternal health services is unclear. The evaluation of this model will thus provide an important contribution to the existing literature.

The study’s contribution to knowledge will be strengthened by certain attributes of the study design. The matched difference-in-difference approach provides better causal inference than that obtained in previous studies of social franchising which have rarely used control groups. The large number of outcomes gives us the opportunity to capture the full range of effects across the continuum of care generated by this multifaceted health system intervention. By accounting for multiple inferences, we deal with the risk of specifying many outcomes. Finally, we complement the impact evaluation with a cost-effectiveness analysis and an examination of the implementation process to understand how the programme worked or failed to work and its potential for scaleup.

The findings will need to be interpreted with several potential limitations in mind. First, we must rely on women to recall delivery care practices during childbirth to come to conclusions as to the impact of the social franchising model on quality of care. Measures of quality that use standardised patients are the gold standard but cannot be used in the case of childbirth [26, 49]. Second, we anticipate issues to do with recall. Some indicators place a heavy burden on the woman’s ability to recollect events up to 2 years ago and are therefore likely to suffer from recall problems. In our adjusted estimates of impact, we control for the recall period and the issue is only a concern for the evaluation insofar as recall bias differs between study arms. Third, we note the potential for bias in our impact estimates given that the offer to join the social franchise network is not randomised. To attempt to limit selection problems, we use matched control areas to provide a credible a counterfactual as possible.

There is a dearth of evidence demonstrating whether market-based approaches such as social franchising can improve care in the private sector. Yet expansion of social franchising models in developing countries has been rapid. There is therefore a critical need for robust evaluations of different social franchising models in a wide range of settings to understand whether they contribute to better population health.

## Additional files

Additional file 1:
**Description of Health Provider Training.** This file provides a detailed description of the health provider training programme, by health provider type. Additional file 2:
**Results Chain of “Sky” Social Franchise Model.** This file provides the results chain of the “Sky” social franchise model and provides a framework for understanding how the programme is intended to work. Additional file 3:
**Demographic and Health Indicators in Intervention Districts of Uttar Pradesh.** This file provides basic demographic and health indicators of the three intervention districts of Uttar Pradesh in which the “Sky” social franchise model is implemented. Additional file 4:
**Empirical Strategy.** This file details the empirical strategy of the impact evaluation.

## Abbreviations

ASHA: Accredited Social Health Activist
AYUSH: Ayurveda, Yoga & Naturopathy, Unani, Siddha, and Homoeopathy
DALY: Disability adjusted life years
GPS: global positioning system
JSY: Janani Suraksha Yojana
LiST: Lives Saved Tool
PHS: Public Healthcare Society
