# Supplementary material for: Protocol for the evaluation of a social franchising model to improve maternal health in Uttar Pradesh, India
Source: Implement Sci. 2015 May 26;10:77. doi: 10.1186/s13012-015-0269-2 (PMC4448271; doi:10.1186/s13012-015-0269-2)
Supplement: Additional file 1: — Description of Health Provider Training. This file provides a detailed description of the health provider training programme, by health provider type. [file 13012_2015_269_MOESM1_ESM.pdf]

Additional File 1: Description of Health Provider Training

Pathfinder International is involved with the training of both public and private providers. Most of the training courses are one time only but there shall also be a Mobile Training and Supervision Unit to ensure post-training follow-up, conduct on-the-job mentoring, and support continuous quality improvement. This team will comprise of the district level health official, a member of the Federation of Obstetric and Gynaecological Societies of India and Pathfinder staff. The training courses are given to various providers as listed below.

Public Sector – Training of public providers will be on general antenatal care, emergency obstetric care (including pre/eclampsia, post-partum haemorrhage), infection prevention, family planning and post-partum contraception counselling. This will involve practical training in the labour room as well:

- Training of Master Trainers: This training is of the obstetric and gynaecological providers from the medical colleges and district hospitals in the intervention districts. These Master Trainers will be the resource pool for the District Level Trainings. This is 3 day training;
- Training of the Medical Officers and Staff Nurses: These will include from all the 29 identified 24x7 facility. An estimated 150 providers shall be trained. This is a three day training in the intervention districts;
- Training of ASHA: This is a one day training at the block level of approximately 2,000 ASHAs. They will be trained on danger signs, birth preparedness, complication readiness and safe delivery, referring women to services, and helping women access insurance schemes.

Private sector – Private providers mainly include obstetric and gynaecological providers at WHP franchised hospitals and AYUSH providers at the SkyHealth centres:

- Training of WHP franchised clinics. All the service providers will be trained on national and international guidelines and protocols for emergency obstetric care (including pre/eclampsia, post-partum haemorrhage), general family planning and post-partum intrauterine device contraception;
- Training of SkyHealth providers. These providers will be trained in general antenatal care, emergency obstetric care (only recognition of complications and timely referrals), family planning and post-partum contraception counselling.
