# Supplementary figures and images for: Protocol for the evaluation of a social franchising model to improve maternal health in Uttar Pradesh, India
Source: Implement Sci. 2015 May 26;10:77. doi: 10.1186/s13012-015-0269-2 (PMC4448271; doi:10.1186/s13012-015-0269-2)

## Additional File 2: Results Chain of “Sky” Social Franchising Model

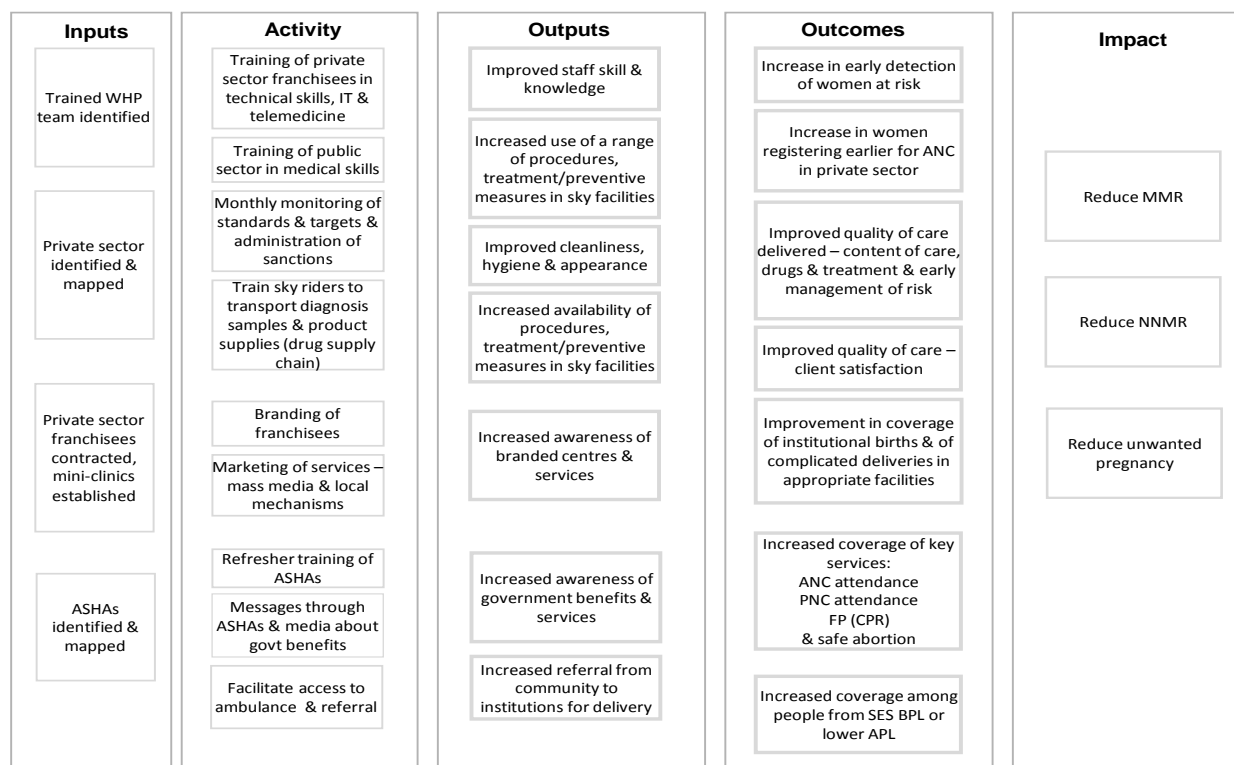

Supplement: Additional file 2: — Results Chain of “Sky” Social Franchise Model. This file provides the results chain of the “Sky” social franchise model and provides a framework for understanding how the programme is intended to work. [file 13012_2015_269_MOESM2_ESM.pdf]
