# Supplementary material for: Protocol for the evaluation of a social franchising model to improve maternal health in Uttar Pradesh, India
Source: Implement Sci. 2015 May 26;10:77. doi: 10.1186/s13012-015-0269-2 (PMC4448271; doi:10.1186/s13012-015-0269-2)
Supplement: Additional file 3: — Demographic and Health Indicators in Intervention Districts of Uttar Pradesh. This file provides basic demographic and health indicators of the three intervention districts of Uttar Pradesh in which the “Sky” social franchise model is implemented. [file 13012_2015_269_MOESM3_ESM.pdf]

Additional File 3: Demographic and health indicators in intervention districts

Indicator	Uttar Pradesh	Kannauj	Kanpur Nagar	Kanpur Dehat
A. Demographic indicators				
Population (in millions)	199.8	1.7	4.6	1.8
Rural population (%)	78	83	34	90
Literacy (%)	57	61	71	65
Fertility (lifetime)	3.3	3.7	2.6	3.2
Maternal mortality ratio (per 100,000)	345	267	267	267
Neonatal mortality (per 1,000)	50	55	24	43
B. Health intervention coverage indicators				
Current use of modern method of family planning	31.8	23.2	39.7	38.6
ANC 3+ visit	29.6	14.5	51.0	32.3
Mothers who received at least TT injection	80.9	78.4	83.0	82.4
Mothers who took IFA more than 100 days	6.5	4.0	19.2	5.8
Mothers whose blood pressure was taken	25.0	12.3	57.2	19.0
Institutional birth rate	45.6	42.4	59.7	47.7
Delivery at home conducted by SBA	21.8	11.2	53.2	28.6
Less than 24 hours stay in institution after delivery	64.7	81.4	41.8	53.0
Mother received post-natal checkup within 48 hours	68.4	48.8	66.5	72.7
Newborn was checked within 24 hours of birth	68.2	49.9	71.7	74.4
Children breastfed within one hour of birth	32.9	27.0	41.0	47.0

Notes: Sources are the Census 2011 and the Annual Health Survey 2010-11. The MMR estimates apply to groups of districts within the state due to sample size limitations. The three intervention districts fall within the same MMR grouping.
