# Supplementary material for: Protocol for the evaluation of a social franchising model to improve maternal health in Uttar Pradesh, India
Source: Implement Sci. 2015 May 26;10:77. doi: 10.1186/s13012-015-0269-2 (PMC4448271; doi:10.1186/s13012-015-0269-2)
Supplement: Additional file 4: — Empirical Strategy. This file details the empirical strategy of the impact evaluation. [file 13012_2015_269_MOESM4_ESM.pdf]

Additional File 4: Empirical Strategy

The impact evaluation relies primarily on the household data. We use a difference-in-difference strategy to estimate impacts [Angrist 2009]. This involves a comparison of changes in the outcomes over time between the intervention and the comparison groups. The analysis exploits the longitudinal nature of the data generated by the recall period used in the two rounds of the household survey and information on the precise timing of the introduction of the social franchising model in each study area.

We test whether the social franchising model has an effect in two ways. The first analysis compares intervention areas with the two sets of controls areas pooled together. The regression takes the form:

$$y_{ict} = \beta_1 SF_{ct} + X_{ict} + \delta_c + \alpha_t + \varepsilon_{ivt} \qquad (1)$$

where y_{ict} is outcome of individual i in study location c in year quarter t, SF_{ct} is a dummy variable indicating whether social franchising has been introduced in the location at the time of birth, X_{ict} is a set of household characteristics, location fixed effects and year quarter fixed effects. Controls for household characteristics include below the poverty line status, urban residence, religion, ethnicity, distance to the nearest government facility, maternal education, parity, multiple birth, and the recall period. We cluster the standard errors at the area level.

The second analysis compares the intervention clusters with those in adjoining districts. Estimates of effect from this analysis may be less prone to selection bias since the comparison clusters are taken from districts that are beyond the geographical reach of the project and hence may offer a more credible counterfactual. The crucial identifying assumption in equation (1) is that, conditional on the controls, introduction of the social franchising programme is orthogonal to the error term. Under this assumption, the coefficient on SF_{ct} captures the causal effect of introducing social franchising on the study outcomes.

We test whether outcomes in the intervention clusters have a trend that is different to those in the comparison areas prior to the introduction of social franchising using a test of pre-trends. For each of the core outcomes, using data from before the introduction of social franchising we estimate a regression of the form:

$$y_{ict} = \beta_1 t + \beta_2 SF \times t + X_{ict} + \delta_c + \varepsilon_{ivt} \qquad (2)$$

where t is the year quarter since the start of the data period, β_1 is the trend in the pooled set of comparison clusters, and β_2 is the difference in the quarterly trend between intervention and control.
